# Carotid Artery Dissection as a Result of Penetrating Ear Trauma

**DOI:** 10.5811/cpcem.2020.6.47537

**Published:** 2020-07-30

**Authors:** Nicholas Peairs, Joel Stillings

**Affiliations:** Desert Regional Medical Center, Department of Emergency Medicine, Palm Springs, California

**Keywords:** Ear trauma, Carotid Artery Dissection

## Abstract

**Case Presentation:**

Here we present the case of a previously healthy 67-year-old female with carotid artery dissection as a result of penetrating ear trauma.

**Discussion:**

Carotid artery dissection can result from unusual mechanisms of injury and present without typical symptoms or exam findings. If left untreated, devastating neurologic sequela can occur. Physicians must have a low threshold to obtain vascular imaging to appropriately manage such cases.

## CASE PRESENTATION

**Image 1:**
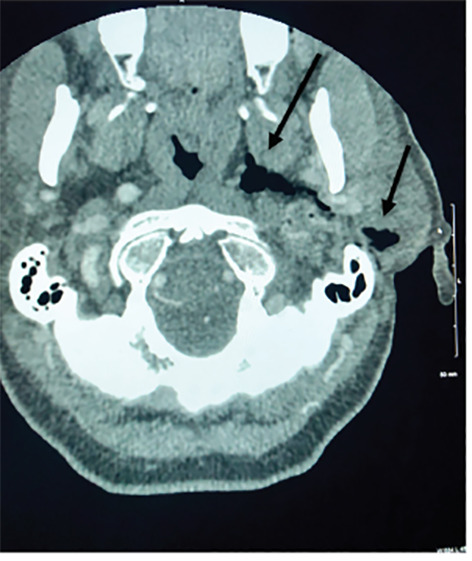
Computed tomography demonstrating soft tissue injury with subcutaneous air extending from the external acoustic meatus to the carotid space (arrows).

A 67-year-old female presented with headache after a four-foot mechanical fall that occurred while hiking. The patient removed a stick from her left ear after a brief loss of consciousness. In the emergency department, the patient had left tongue deviation, a macerated left external acoustic meatus, and hoarse voice. Computed tomography angiography of the head and neck demonstrated soft tissue injury extending inferiorly into the carotid space (Image 1).

**Image 2:**
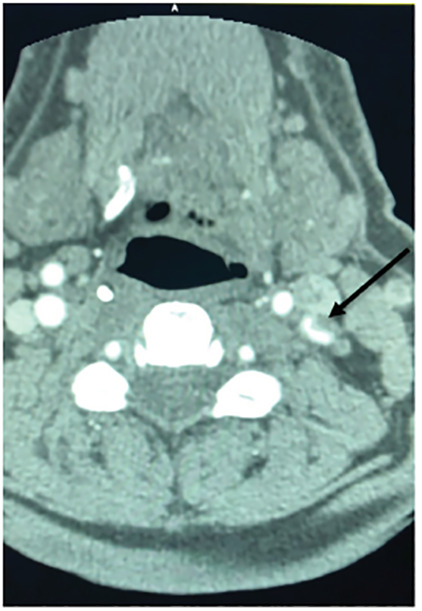
Computed tomography demonstrating dissection of the left internal carotid artery depicting “crescent sign” (arrow).

**Image 3:**
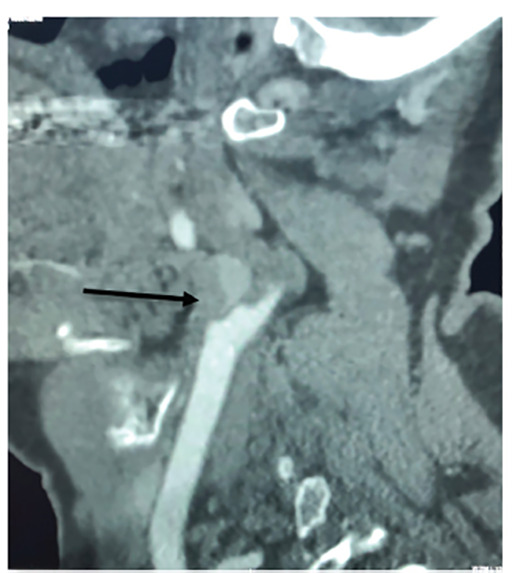
Computed tomography demonstrating complete occlusion of the left internal carotid artery depicting “flame occlusion” (arrow).

Internal carotid artery dissection and occlusion were observed just superior to the carotid bifurcation. Both crescent sign and flame occlusion (Images 2 and 3) are pathognomonic findings on computed tomography angiography neck for carotid dissection and were identified as a result of this trauma. The patient was admitted to the neurological intensive care unit and later discharged with aspirin for stroke prophylaxis and percutaneous endoscopy gastrostomy placement to manage dysphagia from vocal cord paralysis secondary to microvascular injury to the left recurrent laryngeal and hypoglossal nerves.

## DISCUSSION

Classic symptoms of carotid artery dissection include headache, neck pain, and Horner’s syndrome without anhidrosis.^1^ Cerebral ischemia was identified in 67% of patients with carotid artery dissection.^2^ With early detection, antiplatelet prophylaxis can be started to prevent embolism and loss of brain tissue.^3^ Medical providers must consider carotid artery dissection in patients lacking common exam findings and with unusual mechanisms of injury. Rapid diagnostics and intervention are essential to avoid debilitating sequela.

**CPC-EM Capsule**

What do we already know about this clinical entity?

Carotid artery dissection can occur spontaneously or as the result of minor trauma.

What is the major impact of the image(s)?

Penetrating trauma to the external acoustic meatus and disruption of the carotid space has resulted in carotid artery dissection.

How might this improve emergency medicine practice?

Emergency medicine providers must consider carotid artery dissection despite unusual mechanisms of injury and in the absence of common exam findings.
